# Herlyn–Werner–Wunderlich syndrome in a multiparous female

**DOI:** 10.1259/bjrcr.20200132

**Published:** 2020-10-28

**Authors:** HarSumeet Singh Sidhu, Prateek Kumar Madaan

**Affiliations:** 1 Department of Radiodiagnosis, ABVIMS and Dr RML Hospital, New Delhi, India; 2 Department of Radiodiagnosis, VMMC and Safdarjung Hospital, New Delhi, India

## Abstract

Herlyn–Werner–Wunderlich syndrome is a rare complex congenital anomaly of the urogenital tract involving Mullerian ducts and mesonephric ducts. It is also called OHVIRA syndrome (Obstructed Hemivagina and Ipsilateral Renal agenesis). It is characterized by a triad of uterus didelphys, obstructed hemivagina and ipsilateral renal agenesis. Patients usually present after menarche with pelvic pain, dysmenorrhea, mass, and rarely with primary infertility in later years. We report a case of a multiparous female who presented to the hospital with pain in lower abdomen for the past 2 months and acute retention of urine for 1 day. This is an atypical presentation in a multiparous female that has been described in a single case report so far.^1^ Intravenous pyelogram and MRI of the patient revealed uterine didelphys, obstructed right hemivagina causing hematohemicolpos and right renal agenesis. Thorough knowledge of imaging features can enable a radiologist to make a correct diagnosis even in an atypical presentation.

## Clinical presentation

A 31-year-old female presented to the hospital with a chief complaint of acute retention of urine for 1 day. She also complained of pain in the lower abdomen for the past 2 months. The pain was gradual in onset, intermittent, and with episodes of worsening in between. It was radiating to back and lower limbs and not relieved on rest or medication. The patient reported intermittent bleeding during micturition for the past 2 months. There was no history of trauma, instrumentation, or burning micturition. She had no bowel complaints.

The patient attained menarche at 14 years of age and had regular menses since then with average blood flow. She never had complaints of any cyclical dysmenorrhea or any urinary complaints. Her last menstrual period was 20 days ago. She had been married for the last 12 years and was G2P2L2A0 (G-Gravida, P-Parity, L-Live, A-Abortion). Both deliveries were done through Caesarean section due to non-progression of labor in first delivery. The patient was informed regarding the presence of didelphys uterus after first delivery.

**Figure 1.**
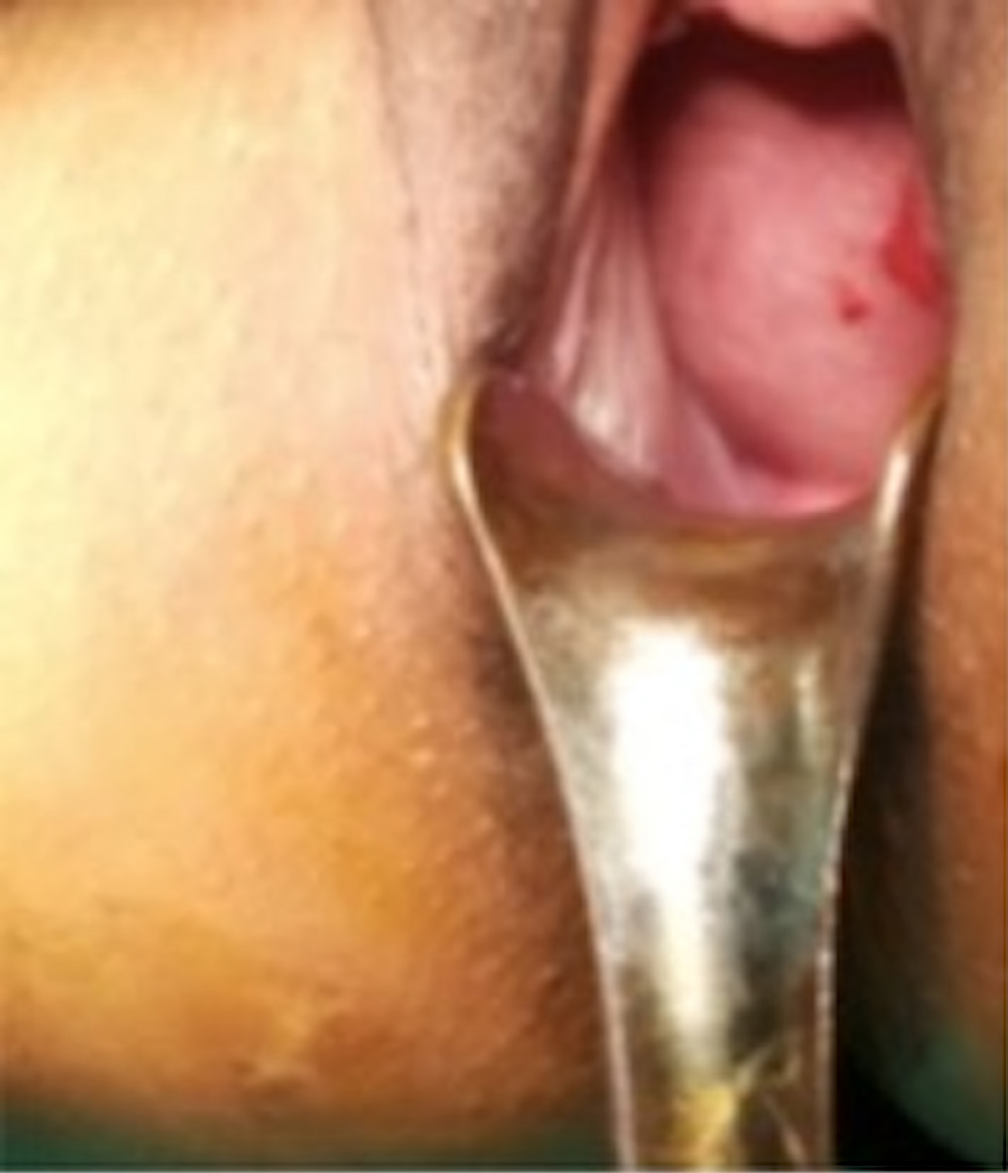
Herlyn-Werner–Wunderlich syndrome in a 31-year-old female. Per speculum examination showed smooth bulge in anterior vaginal –wall caused by hematohemicolpos due to obstructed right hemivagina.

On examination, the patient was conscious and alert. Her vitals were within normal limits. Chest, cardiac, and nervous system examinations were normal. Per abdomen examination revealed soft and tender suprapubic bulge, however, no guarding or rigidity was present. Per speculum examination revealed a large cystic mass in the anterior vaginal wall (Figure 1). Cervical os could not be seen. On per vaginal examination, a large cystic mass was felt occupying the whole of vagina and cervix could not be felt. The patient was catheterized to relieve urinary symptoms.

## Differential diagnosis

Clinical differential diagnoses were ovarian cystic mass and ectopic pregnancy.

## Investigations and imaging findings

All laboratory investigations were within normal limits. Urine pregnancy test was negative.

**Figure 2.**
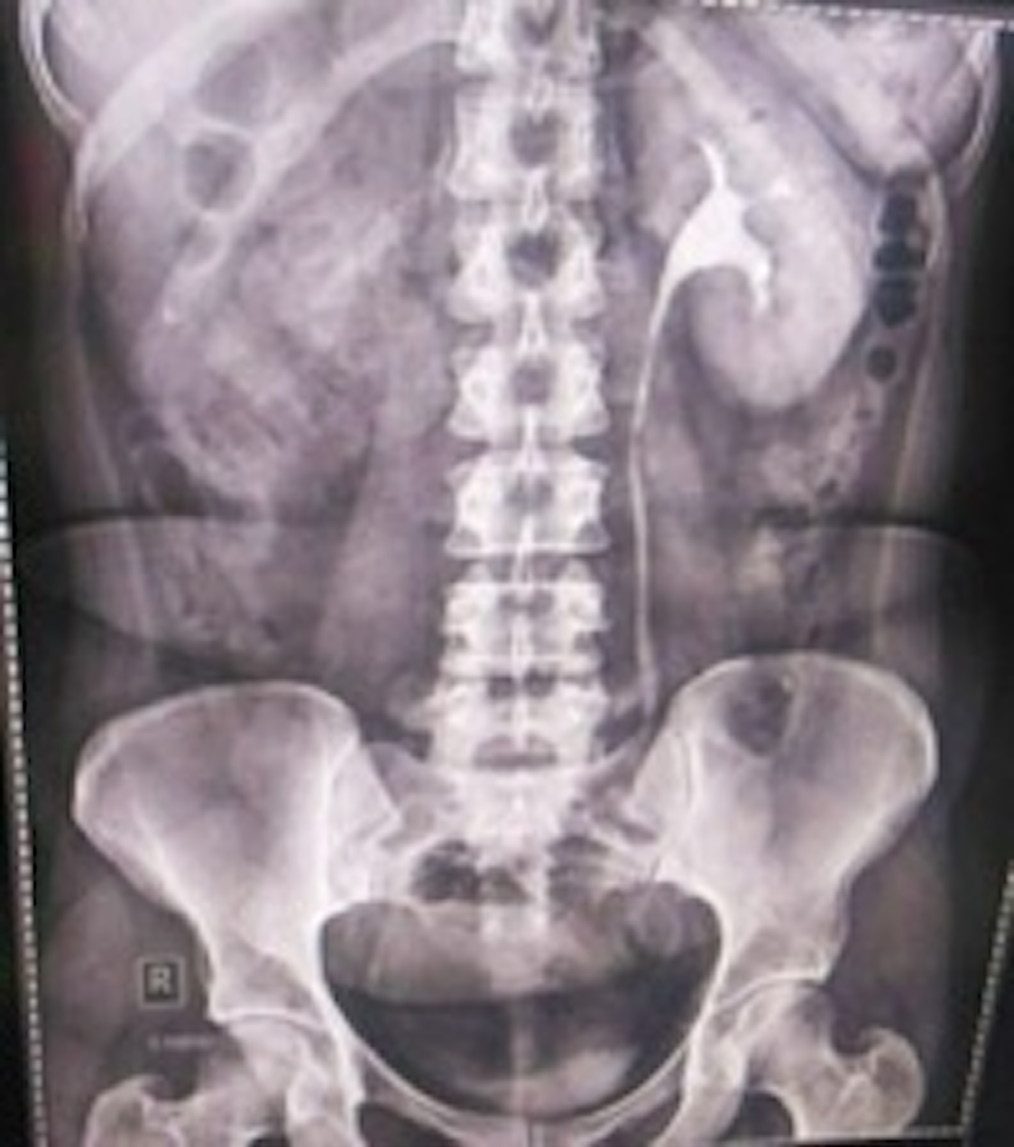
Herlyn–Werner–Wunderlich syndrome in a 31-year-old female. Intravenous pyelogram revealed absent right kidney and normal sized functioning left kidney.

**Figure 3.**
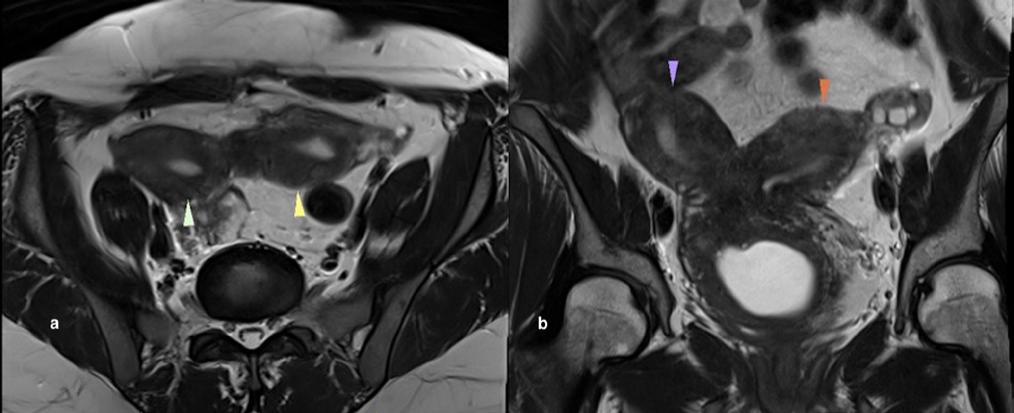
Uterus didelphys. Axial (a) and Coronal (b) *T*_2_ weighted images showed two widely separated normally developed uterine horns (arrowheads).

**Figure 4.**
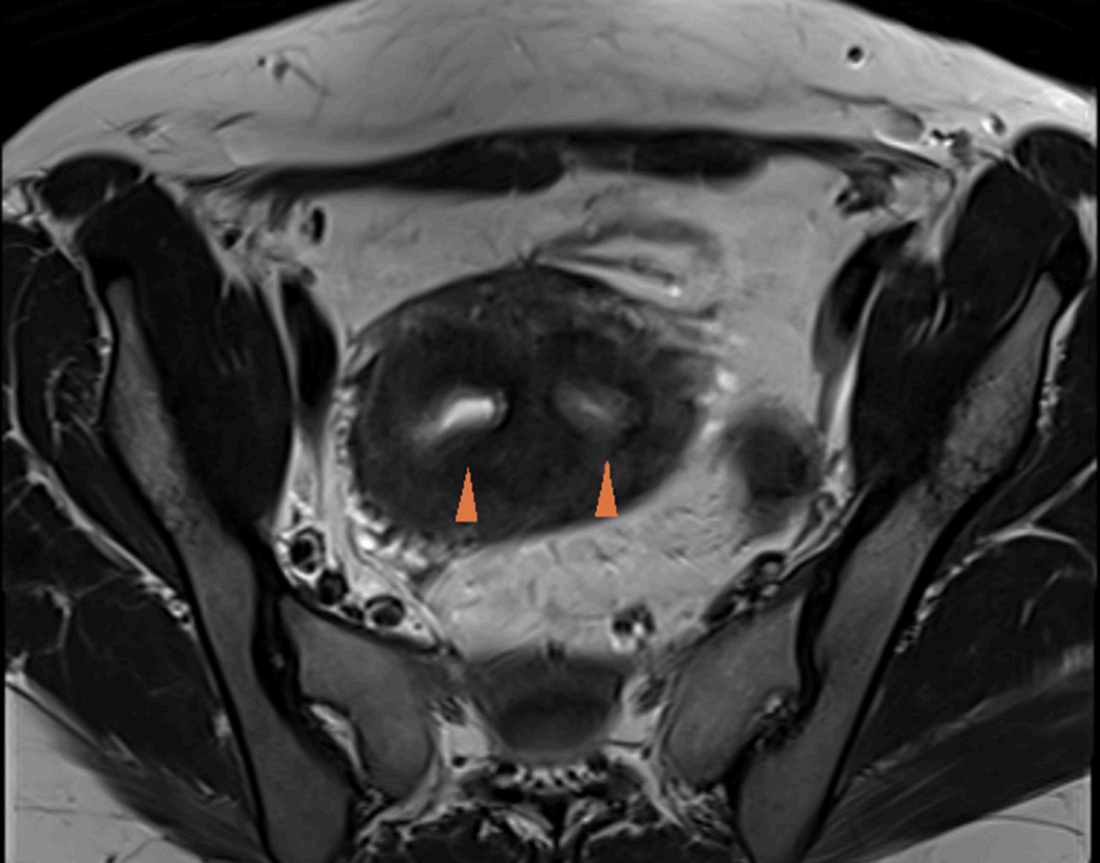
Axial *T*_2_ weighted image showed two separate cervices (arrowheads).

**Figure 5.**
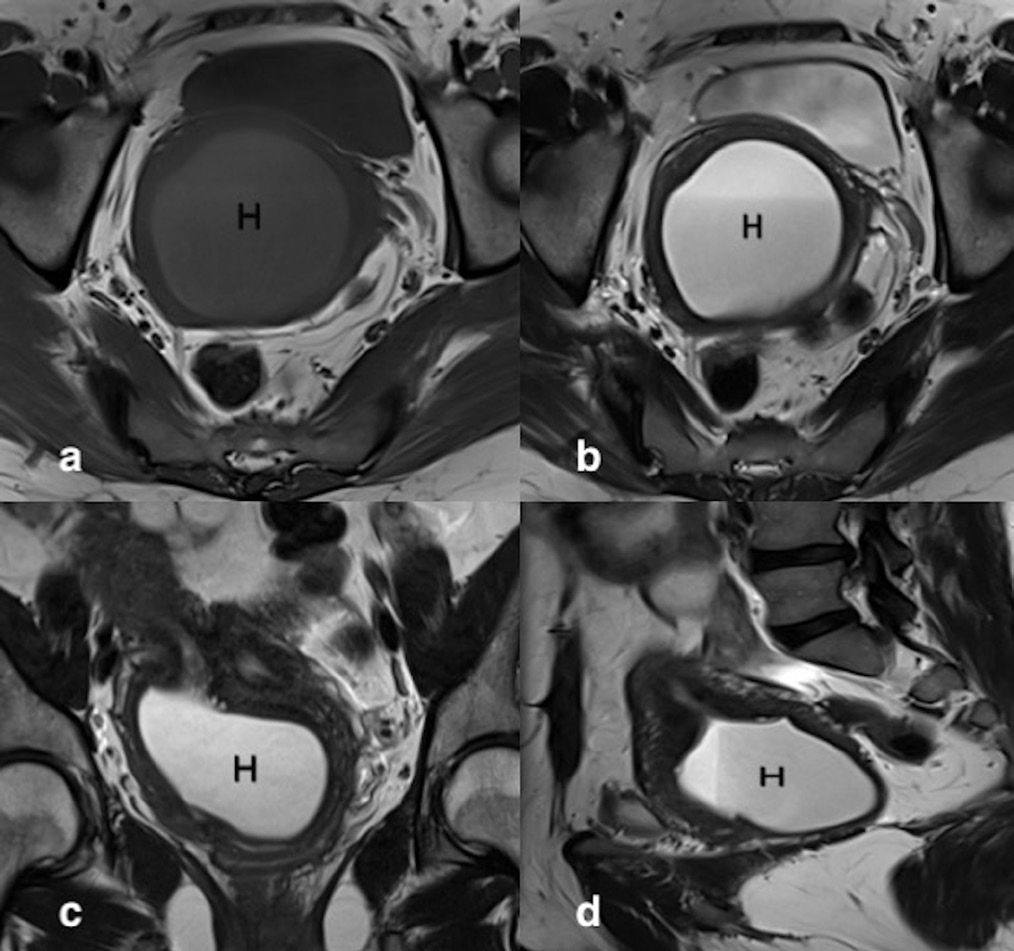
Right hematohemicolpos. Axial *T*_1_ weighted image (a) showed cystic collection (H) with intermediate signal intensity. Axial (b), Coronal (c) and Sagittal (d) *T*_2_ weighted images showed hyperintense signal intensity with fluid-fluid levels.

**Figure 6.**
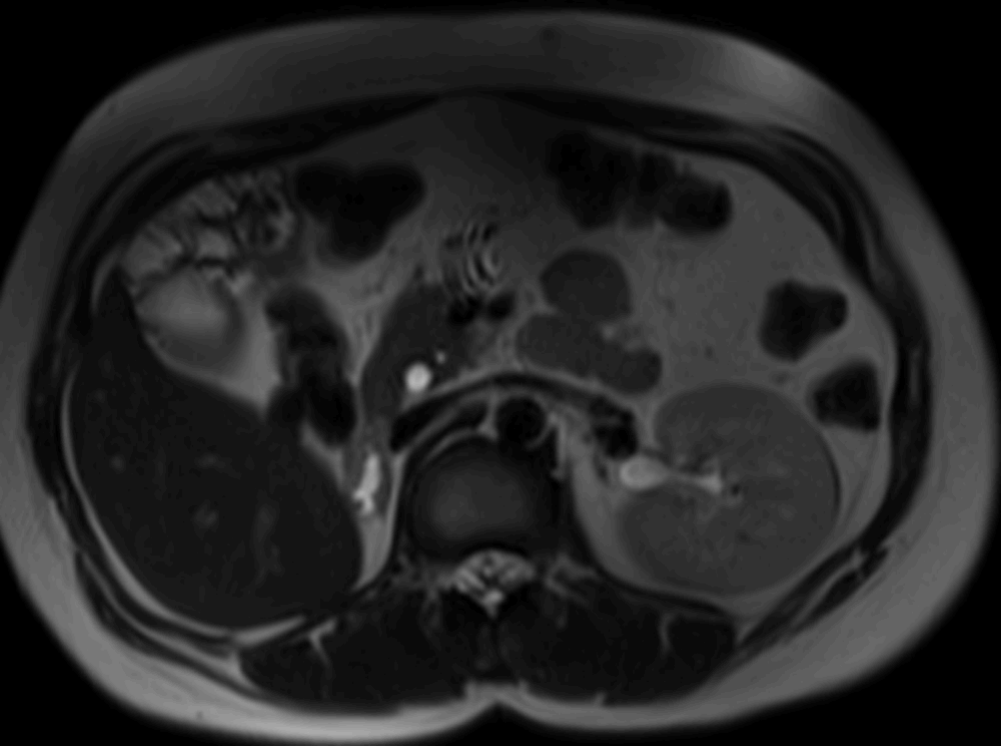
Axial *T*_2_ weighted image of upper abdomen showed absent right kidney and normal left kidney.

Ultrasonography of abdomen and pelvis revealed non-visualization of right kidney, two separate uterine horns, and a cystic collection posterior to uterine horns with low-level echoes within. Intravenous pyelogram revealed the absence of kidney in right renal fossa. No uptake of contrast was seen even after 24 h after intravenous injection of contrast. The left kidney was normal in size, shape, outline, and axis (Figure 2). MRI revealed two widely separated, almost normally developed uterine horns with maintained zonal anatomy, two separate cervices, and patent left vagina (Figures 3 and 4). A cystic collection measuring 7.8 cm x 7.4 cm x 4.9 cm in size showing intermediate signal intensity in *T*_1_ weighted images, hyperintense signal intensity with fluid–fluid levels in *T*_2_ weighted images was noted communicating with the right cervix and located inferior to cervix suggestive of hematohemicolpos (Figure 5). Screening of the abdomen revealed the absence of right kidney (Figure 6). Based on the above-described findings, a provisional diagnosis of uterine didelphys, obstructed right hemivagina causing hematohemicolpos and right renal agenesis was made.

## Treatment, outcome and follow-up

The patient was taken up for laparoscopy which was converted into open laparotomy due to significant adhesions. The findings suggestive of Herlyn–Werner–Wunderlich syndrome on imaging were confirmed. The obstructed right hemivagina and cervix was distended with a collection. Vaginally, a nick was given on the bulge which drained frank pus suggestive of secondary infection of hematocolpos (pyocolpos). The obstructed uterine cavity was opened and a probe, *i.e*. hegar’s dilator was introduced through the cavity. The probe was felt through the vaginal end indicating continuity of cavity with hematocolpos. Right hemihysterectomy was performed to prevent potential complications such as pus collection, recurrence, and endometriosis. The post-operative period was uneventful.

## Discussion

Herlyn–Werner–Wunderlich syndrome is a rare complex congenital anomaly of the urogenital tract that involves abnormal development of the Mullerian and mesonephric ducts in the female embryos.^2–4^ It is also known as OHVIRA syndrome (Obstructed hemivagina and Ipsilateral Renal agenesis). Its estimated occurrence is 0.1–3.8% with only a few reported cases occurring during pregnancy.^5^ It is characterized by uterus didelphys, unilateral obstructed hemivagina, and ipsilateral renal agenesis. The right side is affected twice more frequently than the left side.^6,7^ The syndrome falls under Type III Mullerian duct anomaly classification system of the American Society for Reproductive Medicine (ASRM). According to the newly proposed classification system based on a review of 79 patients, it is categorized as: Classification 1 - a completely obstructed hemivagina (1.1 - with blind hemivagina; 1.2 - cervicovaginal atresia without communicating uteri) and Classification 2 - an incompletely obstructed hemivagina (2.1 – partial resorption of the vaginal septum; 2.2 – with communicating uteri).^8^ Strong suspicion and knowledge of this anomaly are essential for a precise diagnosis.

A typical presentation of this syndrome is dysmenorrhea in adolescent age group, a few months to years after attaining menarche.^9^ Patients can also present with pelvic pain, mass, abnormal vaginal discharge, infertility, endometriosis, complicated pregnancy, and labor.^10^ The initial clinical diagnosis is incorrect in the majority of the cases because of its rare incidence and misleading presenting signs and symptoms. Menstrual flow from the patent unobstructed hemivagina gives the impression of normal menses. Thus, accurate diagnosis and surgical management may be delayed for several months or even years. Our patient had an atypical presentation being an adult female with no previous history of significant dysmenorrhea and obstetric history of two live births by Caesarean section. She presented with acute retention of urine. Such a presentation in a multiparous female has been described in a single case report so far in the literature.^1^

Ultrasonography and MRI are useful in its detection. MRI is the primary modality for diagnosis and pre-operative planning of the Herlyn–Werner–Wunderlich syndrome. It evaluates uterine morphology, detects communication between uterine and vaginal lumen, characterizes fluid contents and diagnose complications like endometriosis.^11^ In our case, MRI findings of two widely separated normally developed uterine horns, two separate cervices, patent left vagina and presence of right hematohemicolpos with associated absent right kidney suggested a pre-operative diagnosis of Herlyn–Werner–Wunderlich syndrome. These findings were subsequently confirmed intraoperatively. As per the newly proposed classification system, considering the late presentation of our case and the intraoperative findings, our case was classified as 2.1 that is incompletely obstructed hemivagina with partial reabsorption of the vaginal septum. The intermittent bloody vaginal discharge that has been described in patients with class 2.1 in the article by Zhu et al^8^ was likely misconstrued as hematuria by the patient in our case.

The management in the typical presentation in adolescent or young females is resection of the vaginal septum followed by vaginoplasty. In our case, right hemihysterectomy was performed since the family of the patient was complete and was not planning for pregnancy in the future.

## Learning points

1. Herlyn–Werner–Wunderlich or OHVIRA syndrome is a rare congenital Mullerian duct anomaly which consists of the presence of uterus didelphys with obstructed hemivagina and ipsilateral renal agenesis.
2. MRI is the investigation of choice for detailed evaluation and classification of Mullerian duct anomaly.
3. Awareness of the imaging features of Herlyn–Werner–Wunderlich syndrome on USG and MRI is a prerequisite for early and prompt diagnosis even in an atypical presentation such as in a multiparous female in our case.
4. Early diagnosis and proper surgical management allow rapid relief of symptoms and prevention of potential complications.

## Patient consent

“Written informed consent was obtained from the patient(s) for publication of this case report, including accompanying images”.
